# Colposcopic Evaluation of Premalignant Lesions of the Cervix and Its Comparison With Liquid-Based Cytology

**DOI:** 10.7759/cureus.112303

**Published:** 2026-07-08

**Authors:** Khushbu Bano, Kalpana Kumari, Pragati Mishra, Shalini Singh, Sarika Pandey

**Affiliations:** 1 Obstetrics and Gynecology, Uttar Pradesh University of Medical Sciences, Etawah, IND

**Keywords:** asccp, ascus, cervical intraepithelial neoplasia (cin), high-grade squamous intraepithelial lesion (hsil), histopathological examination (hpe), human papillomavirus (hpv), liquid-based cytology (lbc), low-grade squamous intraepithelial lesion (lsil)

## Abstract

Background: Cervical cancer remains one of the leading causes of cancer-related mortality in developing countries. Early detection and timely intervention are crucial for reducing both the incidence and mortality associated with cervical cancer. Hence, comparing colposcopy findings with liquid-based cytology (LBC) results in symptomatic women can improve understanding of diagnostic accuracy, guide clinical management, and inform screening strategies.

Materials and methods: A study was conducted in the Department of Obstetrics and Gynecology, Government Medical College, Saifai. This prospective observational study included 180 female participants who fulfilled the predefined inclusion and exclusion criteria. The study group included sexually active women older than 18 years with complaints of discharge per vaginum, postcoital bleeding, postmenopausal bleeding, abnormal vaginal bleeding, or a suspicious cervical abnormality on naked-eye examination. These patients were screened with LBC and colposcopy. Upon detection of any premalignant cervical lesion, a cervical biopsy was performed in 104 participants to confirm the diagnosis by histopathological examination (HPE).

Results: LBC showed a sensitivity of 62.50%, a specificity of 63.88%, a positive predictive value (PPV) of 43.47%, and a negative predictive value (NPV) of 79.31%, suggesting that confirmatory HPE is necessary for an accurate diagnosis. Colposcopy showed a sensitivity of 78.13%, a specificity of 95.83%, a PPV of 89.29%, and an NPV of 90.79%. These results emphasize the importance of both LBC and colposcopy for the effective identification and treatment of cervical lesions.

Conclusion: The study concluded that colposcopy is a more effective modality than LBC for the detection of premalignant cervical lesions.

## Introduction

Cervical cancer is the fourth most common cancer among women worldwide, with an estimated 660,000 new cases and approximately 350,000 deaths reported in 2022. Nearly 94% of these deaths occurred in low- and middle-income countries [1]. Global disparities in cervical cancer burden and mortality are driven by unequal access to vaccination, screening, diagnostic services, and treatment facilities, as well as by socioeconomic determinants such as gender bias, poverty, and the high prevalence of HIV. Women with HIV have a six-fold higher risk of developing cervical cancer. In response to this global health burden, many countries have adopted the World Health Organization (WHO) Global Strategy to eliminate cervical cancer as a public health problem by the year 2030 [1,2].

The WHO Global Strategy targets a reduction in cervical cancer incidence to fewer than four new cases per 100,000 women annually and outlines three targets to be achieved by the year 2030 to put all countries on the pathway to elimination in the coming decades [2]. The three targets are (1) 90% of girls vaccinated with the HPV vaccine by age 15 [2]; (2) 70% of women screened with a high-quality test by ages 35 and 45 [2]; and (3) 90% of women with cervical disease receiving treatment [2].

In India alone, 1,461,427 new cancer cases were reported in 2022, with cervical cancer contributing substantially to the nation's cancer burden and an incidence rate of 100.4 per 100,000 individuals [3]. Traditionally, cervical cancer screening has relied on conventional cytology (Pap smear), supported by colposcopy and histopathological assessment for the detection of premalignant lesions. Biopsy remains the gold standard for confirming cervical lesions [4].

Liquid-based cytology (LBC), a newer cytological technique, has significantly improved sample adequacy, reproducibility, and diagnostic accuracy and has become integral to population-level cervical cancer screening. This method involves suspending exfoliated cervical cells in a liquid medium to create a uniform thin-layer smear, enabling better cellular evaluation [5].

Colposcopy remains a cornerstone of cervical cancer prevention programs. Although initially intended for detecting invasive cervical cancer, its role has expanded to the evaluation of women with abnormal cervical screening results. Colposcopy and biopsy have become important diagnostic tools for women with abnormal cervical screening test results [6]. Colposcopy is a diagnostic procedure performed following an abnormal cervical screening test or the identification of a visible cervical lesion during examination. This procedure assists in formulating a management plan based on the biopsy results. Generally, lesions can be observed or treated according to evidence-based guidelines. Low-grade lesions can be followed up and managed according to the American Society for Colposcopy and Cervical Pathology (ASCCP) guideline algorithms. High-grade lesions are treated depending on the patient's age and fertility status [7].

## Materials and methods

This study was conducted among outpatients in the Department of Obstetrics and Gynecology, Uttar Pradesh University of Medical Sciences (UPUMS), Saifai, Etawah, over a period of 18 months from October 2022 to March 2024. The study population included 180 women who met the predefined inclusion and exclusion criteria. All participants were older than 18 years, sexually active, and presented with complaints of postmenopausal bleeding, discharge per vaginum, postcoital bleeding, irregular vaginal bleeding, or a suspicious cervical abnormality on naked-eye examination. Participants were selected randomly from the gynecology outpatient department (OPD).

The inclusion criteria included sexually active women older than 18 years with complaints of postcoital bleeding, vaginal discharge, abnormal vaginal bleeding, postmenopausal bleeding, or a suspicious cervical abnormality, and who provided informed consent to participate in the study.

The exclusion criteria included women younger than 18 years, unmarried women, women who were pregnant or in the immediate postpartum period, women with acute cervical infection, women with active vaginal bleeding, and women who refused to participate in the study.

Statistical analysis

The study proforma was completed, and the data were collected. Data were entered into Microsoft Excel 2019 and analyzed using SPSS version 21 (IBM Corp., Armonk, NY) with appropriate standard statistical methods. Continuous variables were summarized as mean±SD, whereas categorical variables were expressed as frequencies and percentages. Associations between categorical variables were assessed using the Pearson χ² test. Comparisons of mean values were performed using the Student's t-test. Histopathological examination (HPE) was considered the gold standard for evaluating the diagnostic performance of LBC and colposcopy.

Following approval from the Institutional Ethical Clearance Committee, written informed consent was obtained from all participants. Sociodemographic data, including age, marital status, religion, education, occupation, and socioeconomic status, were collected using a standardized questionnaire. A detailed clinical history was obtained, including presenting complaints, menstrual and obstetric history, and other relevant medical information. Each participant underwent a general physical examination, during which vital parameters, including heart rate, respiratory rate, blood pressure, and temperature, were recorded, followed by a systemic clinical assessment. A pelvic examination was conducted using a sterile speculum to inspect for cervical discharge, erosion, or other abnormalities. A sample for LBC was collected first, followed by a colposcopy examination during the same session. In cases with abnormal colposcopy findings, a directed cervical biopsy was performed for histopathological confirmation.

Collection of specimens for LBC

Following placement of the patient in the lithotomy position, a Cusco's speculum was introduced into the vaginal canal to visualize the cervix. The cervical area was cleaned using sterile normal saline. Samples were collected from the ectocervix and endocervix using a Rover's Cervex-Brush. The brush head was then detached and placed into a BD SurePath™ vial containing a 24% ethanol-based preservative solution. This medium served as a fixative and antimicrobial transport solution for gynecological specimens. The sample was sent to the Department of Pathology, UPUMS, Saifai, Etawah, Uttar Pradesh, for processing and cytological evaluation. Interpretation of results was performed according to the 2001 Bethesda System guidelines for cervical/vaginal cytological diagnosis.

Procedure for colposcopy

Colposcopy was performed in all symptomatic women. After insertion of a self-retaining Cusco's speculum, the cervix was visualized, and any mucus was removed using a saline-moistened cotton swab. A green filter was used to enhance visualization of cervical vascular patterns, aiding in the identification of changes such as punctuation, mosaic pattern, and irregular branching vessels, which may indicate precancerous lesions. The cervix was examined three minutes after the application of a freshly prepared 3% acetic acid solution. After three minutes, areas with acetowhite changes were evaluated, followed by the application of Lugol's iodine, and the findings were assessed using the Modified Reid's Colposcopy Index (Table 1 and Table 2) [8,9]. Of the 180 patients enrolled in the study, HPE was performed in 104 patients with premalignant cervical lesions who underwent cervical biopsy. Specimens were preserved in either LBC medium or 10% neutral-buffered formalin, depending on the specimen type. All samples were submitted to the Department of Pathology, UPUMS, Saifai, for histopathological analysis. The remaining 76 patients did not undergo biopsy because of normal screening findings, loss to follow-up, or refusal to provide consent for the procedure. These participants were excluded from histopathological verification for ethical and practical reasons. Histopathological findings from the 104 patients were used as the reference standard for evaluating the diagnostic performance of LBC and colposcopy.

**Table 1:** Modified Reid’s colposcopy index AWA, acetowhite area Source: [8]

Colposcopy sign	Score 0	Score 1	Score 2
Margin	Condylomatous or micropapillary contour; flocculated or feathered, jagged, angular, satellite lesion; AWA beyond the squamocolumnar junction.	Regular lesion with smooth, indistinct borders	Rolled, peeling edges; sharp margins
Color	Shiny, snow-white area of faint (semitransparent) whitening	Intermediate shade (shiny but gray-white)	Dull, oyster-gray
Vessels	Uniform, fine-caliber, nondilated capillary loops; fine punctuation or mosaic	Absence of surface vessels	Definite coarse punctuation or mosaic
Iodine staining	Any lesion staining mahogany brown; mustard-yellow staining in a minor lesion (based on the first three criteria)	Partial iodine uptake (mottled pattern)	Mustard-yellow staining of a significant lesion (an acetowhite area scoring 3 or more points based on the first three criteria)

**Table 2:** Reid's colposcopic score The severity of cervical lesions was graded using Reid's Colposcopic Index. HPV, human papilloma virus; CIN, cervical intraepithelial neoplasia Source: [9]

Score	Colposcopic findings
0-2	Low-grade disease (likely CIN 1)
3-5	Intermediate-grade disease (likely CIN 1-CIN 2)
6-8	High-grade disease (likely CIN 2-CIN 3)

## Results

**Figure 1:**
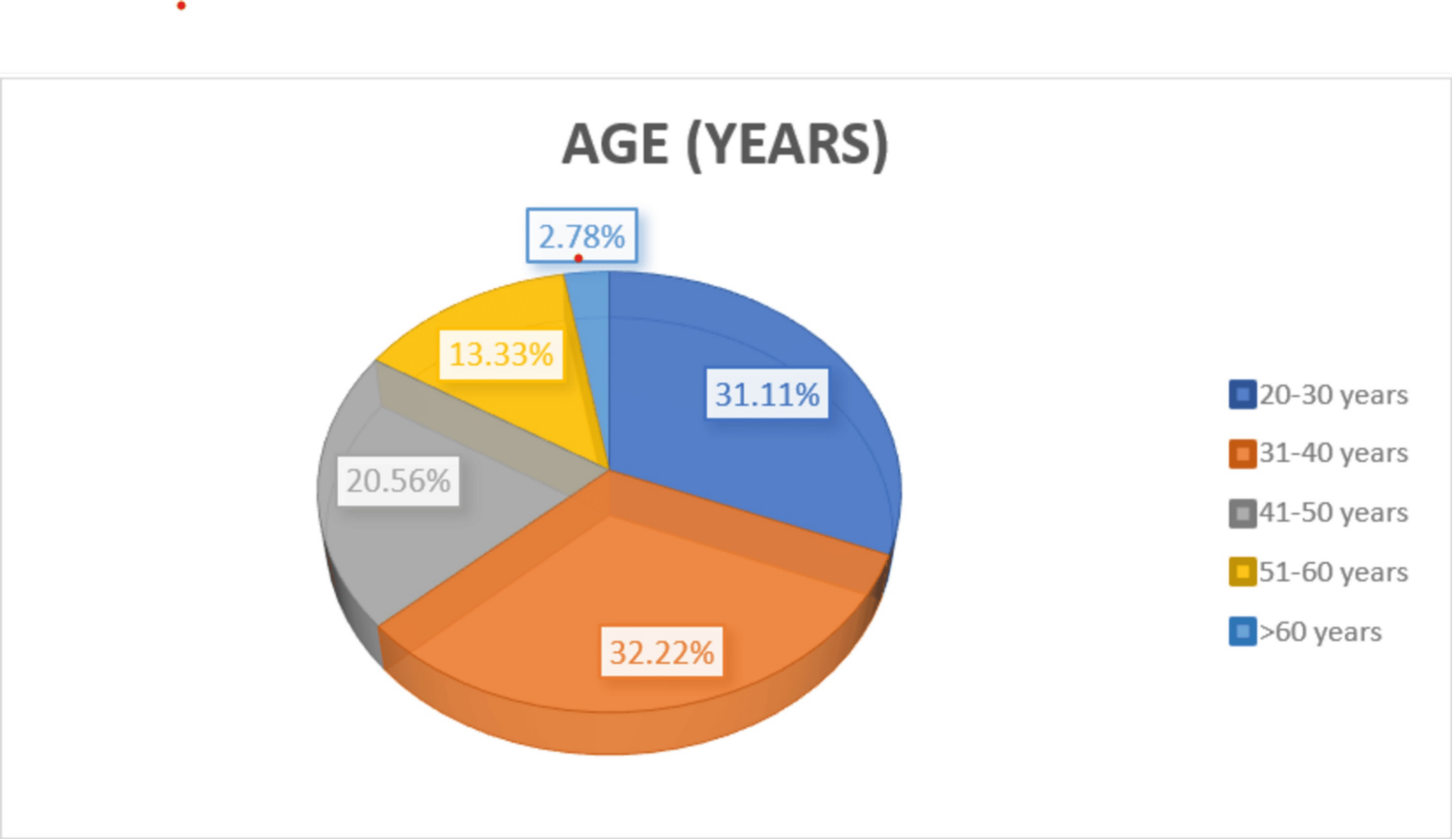
Distribution of patients by age group (years)

In this study, the age distribution of 180 symptomatic women was analyzed. The majority of patients fell within the 31-40-year age group, comprising 32.22% (n=58) of the sample (Figure 1). This was followed by the 20-30-year group at 31.11% (n=56). Women aged 41-50 years accounted for 20.56% (n=37), while those aged 51-60 years made up 13.33% (n=24). Only 2.78% (n=5) were over 60 years. The mean age of the participants was 39.06±11.83 years, indicating a diverse age range among the symptomatic women studied (Table 3).

**Table 3:** Distribution of patients according to age

	Age (years)	n=180	%
Age (years)	20-30 years	56	31.11
	31-40 years	58	32.22
	41-50 years	37	20.56
	51-60 years	24	13.33
	>60 years	5	2.78
	Mean±SD	39.06±11.83	

**Figure 2:**
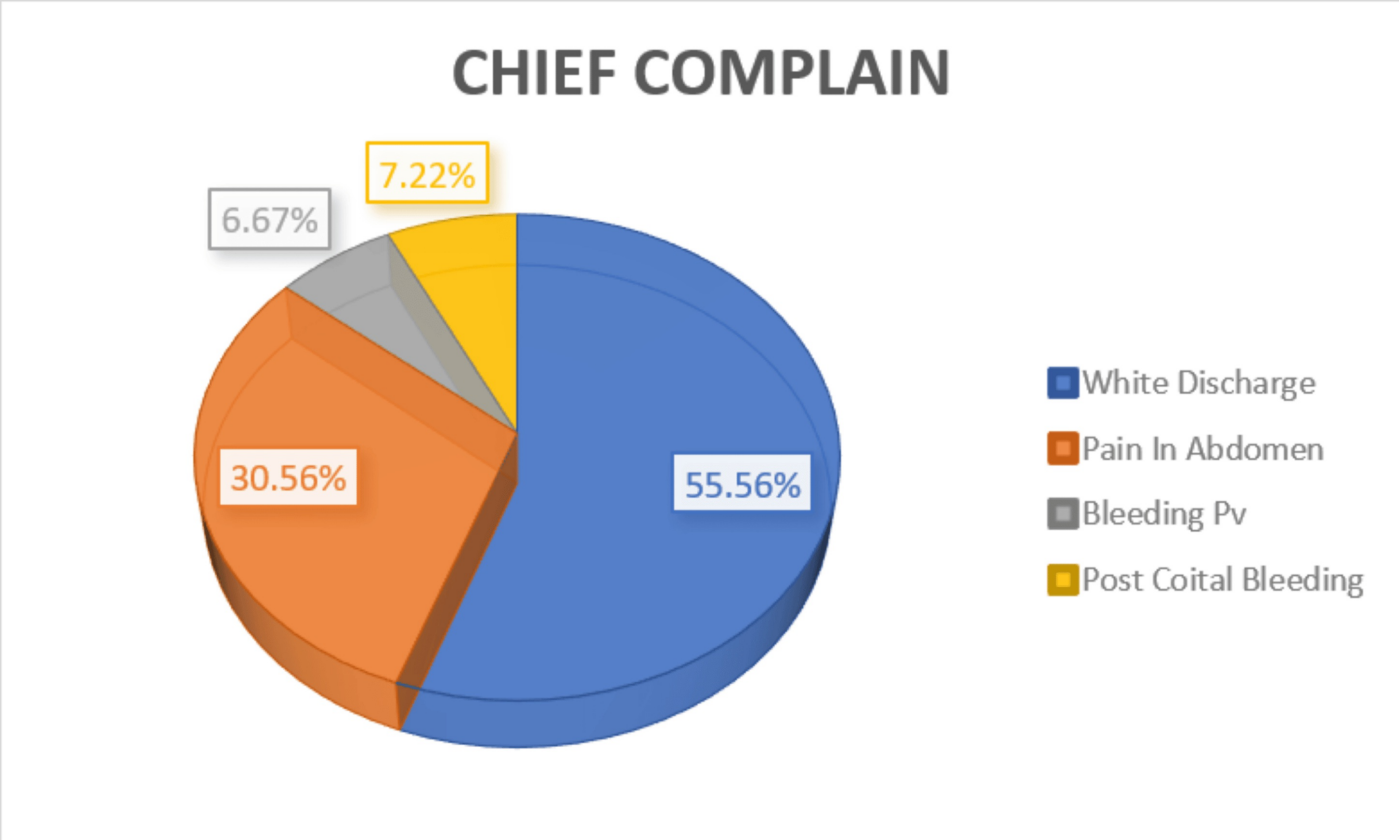
Distribution of patients according to chief complaint PV, per vaginum

The most common complaint was white discharge, reported by 55.56% (n=100) of the participants. Abdominal pain was the second most frequent complaint, affecting 30.56% (n=55) of the women. Postcoital bleeding was reported by 7.22% (n=13) of the patients, while 6.67% (n=12) experienced bleeding per vaginum (Figure 2). These findings underscore the range of symptoms experienced by women with cervical pathology, highlighting the need for comprehensive diagnostic evaluation.

The most common per speculum finding was discharge, observed in 51.11% (n=92) of the patients. No findings were reported in 39.44% (n=71) of the cases. Erosion was noted in 9.44% (n=17) (Table 4). These results highlight the prevalence of discharge as a primary finding in women undergoing per speculum examination.

**Table 4:** Distribution of patients according to per-speculum findings

	Finding	n=180	%
Per speculum	No findings	71	39.44
	Discharge	92	51.11
	Erosion	17	9.44

The majority of patients, 39.44% (n=71), had negative for intraepithelial lesion or malignancy (NILM). Inflammatory results were observed in 35% (n=63) of the cases. Atypical squamous cells of undetermined significance (ASCUS) were observed in 9.44% (n=17), while low-grade squamous intraepithelial lesions (LSIL) were present in 7.78% (n=14). High-grade squamous intraepithelial lesions (HSIL) were found in 3.89% (n=7), atypical squamous cells, cannot exclude a high-grade squamous intraepithelial lesion (ASC-H), in 3.33% (n=6), and squamous cell carcinoma (SCC) in 1.11% (n=2) (Table 5).

**Table 5:** Distribution of patients according to LBC reports NILM, negative for intraepithelial lesion or malignancy; ASCUS, atypical squamous cells of undetermined significance; LSIL, low-grade squamous intraepithelial lesion; HSIL, high-grade squamous intraepithelial lesion; ASC-H, atypical squamous cells, cannot exclude high-grade squamous intraepithelial lesion; SCC, squamous cell carcinoma; LBC, liquid-based cytology

	Category	Frequency (n=180)	Percentage (%)
LBC	NILM	71	39.44
	ASCUS	17	9.44
	LSIL	14	7.78
	Inflammatory	63	35.00
	HSIL	7	3.89
	ASC-H	6	3.33
	SCC	2	1.11

The majority of patients, 82.22% (n=148), had a Reid score of 0-2. This was followed by 13.33% (n=24) with a Reid score of 3-5. A total of 4.44% (n=8) had a Reid score of 6-8 (Table 6). These findings help in understanding the severity and distribution of cervical abnormalities among the participants.

**Table 6:** Distribution of patients according to Reid colposcopy score

	Reid score	n=180	%
Colposcopy Reid score	0-2	148	82.22
	3-5	24	13.33
	6-8	8	4.44

**Figure 3:**
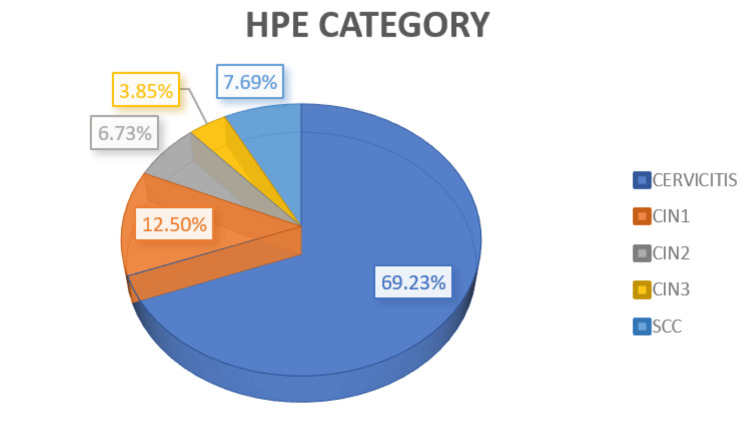
Distribution of patients according to HPE report HPE, histopathological examination; CIN, cervical intraepithelial neoplasia; SCC, squamous cell carcinoma

Of the 180 women enrolled, 104 underwent cervical biopsy with HPE. The majority, 69.23% (n=72), were diagnosed with cervicitis. The distribution of cervical intraepithelial neoplasia (CIN) categories was as follows: CIN 1 was found in 12.50% (n=13), CIN 2 in 6.73% (n=7), and CIN 3 in 3.85% (n=4). SCC was found in 7.69% (n=8) of the patients (Figure 3). These results suggest that cervicitis was the most common diagnosis, with a considerable proportion of patients also showing precancerous lesions and invasive malignancy. 

Agreement between LBC and biopsy was assessed, and the results are shown in Table 7. A p-value of 0.012 was obtained using the chi-square test, indicating a statistically significant association between LBC and biopsy (Table 8).

**Table 7:** Agreement between LBC and biopsy LBC, liquid-based cytology; NILM, negative for intraepithelial lesion or malignancy; ASCUS, atypical squamous cells of undetermined significance; ASC-H, atypical squamous cells, cannot exclude high-grade squamous intraepithelial lesion; LSIL, low-grade squamous intraepithelial lesion; HSIL, high-grade squamous intraepithelial lesion; SCC, squamous cell carcinoma

LBC	Chronic cervicitis	CIN 1	CIN 2	CIN 3	SCC	Total
NILM, inflammatory (n=58)	46	7	3	1	1	58
ASCUS, ASC-H (n=23)	16	3	2	1	1	23
LSIL (n=14)	9	2	1	1	1	14
HSIL (n=7)	1	1	1	1	3	7
SCC (n=2)	0	0	0	0	2	2
Total	72	13	7	4	8	104

**Table 8:** Evaluation of LBC for CIN LBC, liquid-based cytology; CIN, cervical intraepithelial neoplasia

LBC	Biopsy positive	Biopsy negative	Total
Positive	20 (true positive)	26 (false positive)	46
Negative	12 (false negative)	46 (true negative)	58
Total	32	72	104
P-value	0.012		

In this study, the diagnostic performance of LBC for detecting premalignant cervical lesions was assessed against HPE findings. LBC demonstrated a sensitivity of 62.50% and a specificity of 63.88%, missing 12 of the 32 biopsy-positive cases. The positive predictive value (PPV) was 43.47%, and the negative predictive value (NPV) was 79.31% (Table 9). These results suggest that while LBC is useful for initial screening, its relatively low specificity necessitates confirmatory testing with HPE.

**Table 9:** Sensitivity, specificity, PPV, and NPV of LBC LBC, liquid-based cytology; PPV, positive predictive value; NPV, negative predictive value

	Sensitivity and 95% CI	Specificity and 95% CI	PPV and 95% CI	NPV and 95% CI
LBC	62.50% (95% CI: 45.1%-79.3%)	63.88% (95% CI: 52.8%-74.9%)	43.47% (95% CI: 29.2%-57.8%)	79.31% (95% CI: 68.9%-89.7%)

Agreement between colposcopy and biopsy was assessed, and the results are shown in Table 10. A p-value of <0.0001 indicated a highly statistically significant association between the two modalities (Table 11).

**Table 10:** Agreement between colposcopy and biopsy CIN, cervical intraepithelial neoplasia; SCC, squamous cell carcinoma

Reid score	Chronic cervicitis	CIN 1	CIN 2	CIN 3	SCC	Total
0-2 (n=76)	69	4	1	1	1	76
3-5 (n=20)	3	6	6	2	3	20
6-8 (n=8)	0	3	0	1	4	8
Total	72	13	7	4	8	104

**Table 11:** Evaluation of colposcopy for CIN CIN, cervical intraepithelial neoplasia

Colposcopy	Biopsy positive	Biopsy negative	Total
Positive	25 (true positive)	3 (false positive)	28
Negative	7 (false negative)	69 (true negative)	76
Total	32	72	104
P-value	<0.00001		

The colposcopic Reid score demonstrated high diagnostic accuracy for detecting premalignant cervical lesions when compared with HPE findings. It had a sensitivity of 78.13%, a specificity of 95.83%, a PPV of 89.29%, and an NPV of 90.79% (Table 12). Overall, the Reid score is a reliable diagnostic tool.

**Table 12:** Sensitivity, specificity, PPV, and NPV of colposcopy PPV, positive predictive value; NPV, negative predictive value

	Sensitivity and 95% CI	Specificity and 95% CI	PPV and 95% CI	NPV and 95% CI
Colposcopy Reid score	78.13% (95% CI: 63.6%-92.7%)	95.83% (95% CI: 91.2%-100.0%)	89.29% ( 95% CI: 77.8%-100.0%)	90.79% (95% CI: 84.3%-97.3%)

**Figure 4:**
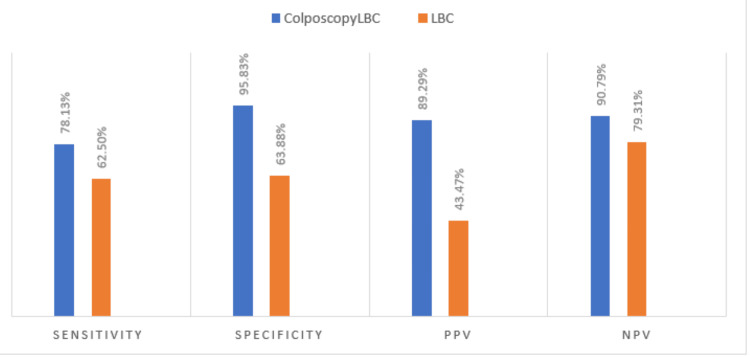
Comparison between colposcopy and LBC LBC, liquid-based cytology; PPV, positive predictive value; NPV, negative predictive value

Colposcopy demonstrated better diagnostic performance than LBC, with a sensitivity and specificity of 78.13% and 95.83%, respectively, compared with 62.50% and 63.88% for LBC. The PPV and NPV of colposcopy were 89.29% and 90.79%, respectively, whereas the PPV and NPV of LBC were 43.47% and 79.31%, respectively (Figure 4). The agreement between LBC and colposcopy was evaluated in 104 participants. A p-value of 0.006, calculated using the chi-square test, indicated a highly significant association. The kappa coefficient was 0.21, indicating fair agreement between LBC and colposcopy.

## Discussion

Colposcopy and cytology are crucial diagnostic tools in the detection and management of cervical abnormalities, especially in symptomatic women. Understanding the correlation between these methods is essential for optimizing patient care and improving the accuracy of cervical cancer screening programs.

The present study was conducted to evaluate the cervix of symptomatic women by colposcopy and its correlation with LBC. To establish LBC and colposcopy as preferred and more accurate diagnostic methods, the aim was to minimize the need for repeat clinical visits and dependence on invasive procedures such as biopsies.

Prasad et al. found a significant prevalence of cervical abnormalities in the 31-40-year age group, which was also observed in the majority of patients (32.22%, n=58) [10]. The proportion of women aged 41-50 years was 20.56% (n=37), which is consistent with the findings of Kalyankar et al. [11]. Kumari et al. found that the mean age of their patients was 36±3 years [12]. These results highlight the need for tailored cervical screening programs for different age groups to effectively detect and reduce cervical abnormalities.

In this study, the predominant complaint was the presence of white discharge, which was noted by 55.56% (n=100) of women. This finding is similar to the findings of Garg et al., who observed that 58.5% of women with cervical abnormalities had white discharge [13]. Prasad et al. documented white discharge in 50% of symptomatic women, and Kalyankar et al. reported white discharge in 56.12% of patients [10,11]. Abdominal discomfort, the second most common symptom in our study, was noted by 30.56% (n=55) of the participants. Prasad et al. found that 20% of symptomatic women had pelvic pain, while Kalyankar et al. reported abdominal discomfort in 16.32% of patients [10,11].

In our study, discharge was the predominant finding in 51.11% of patients on per speculum examination. This finding is similar to the findings of Prasad et al., who found that discharge was a notable symptom in 50% of symptomatic women who required colposcopic examination [10]. Similarly, Garg et al. found that 58.5% of women had discharge [13].

Most patients in our study had a NILM. Among them, 35% showed inflammation, which is consistent with the findings of Garg et al., who also observed significant inflammatory changes [13]. Similarly, Patel et al. reported the presence of inflammatory smears in 58% of cases [14]. ASCUS was detected in 9.44% of our patients, which is consistent with the findings of Prasad et al. and Patel et al. [13,14]. ASC-H was found in 3.33% of our study participants, consistent with the findings of Prasad et al., and this underscores the need for thorough follow-up [10]. LSIL was detected in 7.78% of patients, which is consistent with the findings of Kalyankar et al. and Patel et al. and highlights the need for appropriate screening [11-14]. A total of 3.89% of cases were found to have HSIL. These findings indicate the need for immediate treatment [10,11], as noted by Prasad et al. and Garg et al [10,13].

In our study, 104 of the 180 symptomatic women had HPE reports. The incidence of cervicitis, CIN I, CIN II, and CIN III is consistent with the findings of Kumari et al., who documented cervicitis in 34.7% of their subjects [12]. The high percentage of individuals with CIN I (12.50%) is consistent with the findings of Kalyankar et al. [11].

In our study, the sensitivity of LBC was determined to be 62.50%, the specificity was 63.88%, the PPV was 43.47%, and the NPV was 79.31%. These findings suggest that while LBC is beneficial for initial screening, confirmatory testing remains necessary. Different studies comparing the evaluation of LBC are presented in Table 13.

**Table 13:** Comparison of different studies evaluating LBC LBC, liquid-based cytology

Author and year of study	Age group in years	Sensitivity	Specificity
Prasad et al. [10]	31-40	91.7%	45.45%
Kumari et al. [12]	33-40	87.7%	78.2%
Garg et al. [13]	-	44%	-
Kamiri-Zarchi et al. [15]	21-70	55.3%	77.7%
Barrios et al. [16]	25-52	76.5%	66.0%
Singh et al. [17]	18-65	67.25%	64.9%
Rajkumar et al. [18]	-	62.89%	89.32%
Balwinder Kaur et al. [19]	21-40	61.70%	55.10%
Present study	20-60	62.50%	63.88%

The Reid Score is a reliable diagnostic tool. The high sensitivity of the Reid Score, as demonstrated by Prasad et al., underlines its effectiveness in identifying cervical abnormalities and minimizing cases of misdiagnosis [10]. The specificity of the method enhances its ability to reduce false-positive results, which is essential for patient care, as emphasized by Garg et al. [13]. The PPV and NPV demonstrate the reliability of clinical decision-making and are consistent with the findings of Kumari et al. and Patel et al. [12-14]. These studies confirm the effectiveness of the Reid Score in improving diagnostic accuracy and patient outcomes in cervical cancer screening initiatives. Different studies comparing the evaluation of colposcopy are presented in Table 14.

**Table 14:** Comparison of different studies evaluating colposcopy

Author and year of study	Age group in years	Sensitivity	Specificity
Prasad et al. [10]	31-40	83.37%	72.72%
Kumari et al. [12]	33-40	89.4%	98.8%
Garg et al. [13]	-	88%	-
Kamiri-Zarchi et al. [15]	21-70	70.90%	44.4%
Singh et al. [17]	18-65	90.3%	97.7%
Rajkumar et al. [18]	-	70%	91.26%
Balwinder Kaur et al. [19]	21-40	80.85%	96.93%
Present study	20-60	78.13%	95.83%

The study conducted by Kalyankar et al. provided evidence that, in women with abnormal LBC, colposcopic results showed the presence of CIN I (23.2%), CIN II (9.1%), and CIN III (2.6%) [12]. These findings suggest a similar spectrum of cervical abnormalities to that observed in our study. This is consistent with the distribution of Reid scores observed in our study and emphasizes the high incidence of significant cervical abnormalities requiring medical attention. The comprehensive analysis by Kalyankar et al. emphasizes the effectiveness of colposcopy in verifying cytological observations, which is essential for accurate diagnosis and treatment planning [12]. The agreement between our study and previous studies suggests that colposcopy is a better modality for the effective management of premalignant cervical lesions.

## Conclusions

This study highlights the effectiveness of LBC and the Reid colposcopy score in detecting early cervical cancer. The sensitivity of LBC was 62.50%, indicating its effectiveness as an initial screening method. However, the specificity was 63.88%, suggesting that confirmatory testing with HPE is necessary for accurate diagnosis. On the other hand, the Reid colposcopy score showed excellent diagnostic accuracy, with a sensitivity of 78.13% and a specificity of 95.83%. However, because HPE confirmation was available only for a subset of participants, the findings should be interpreted with caution due to the potential for partial verification bias. Larger multicenter prospective studies with complete histopathological verification are needed to confirm these results. These results emphasize the importance of comprehensive screening methods, including both LBC and colposcopy, to ensure the efficient identification and treatment of premalignant cervical lesions. The validity of using the Reid score as a diagnostic tool is supported by these findings, as it improves the accuracy of detecting both true-positive and true-negative cases in cervical cancer screening programs, leading to better patient outcomes.
